# Letter to the editor: Vitamin D levels in acute illness and clinical severity in COVID-19 patients

**DOI:** 10.1186/s12931-021-01703-1

**Published:** 2021-04-09

**Authors:** Prateek Lohia, Shweta Kapur, Pragnesh Patel, Berhane Seyoum

**Affiliations:** 1 Department of Internal Medicine, Wayne State University, 4201 St Antoine, UHC 5C, Detroit, MI 48201 USA; 2 Wayne State University, Detroit, MI USA; 3 Department of Geriatrics, Wayne State University, Detroit, MI USA; 4 Department of Endocrinology, Wayne State University, Detroit, MI USA

**Keywords:** COVID-19, SARS-CoV-2, Vitamin D, Inflammation, SOFA, Mortality, 25-Hydroxyvitamin D, Intensive care, Mechanical ventilation, Interleukin-6

## Abstract

We would like to comment on the recently published article titled: “Circulating Vitamin D levels status and clinical prognostic indices in COVID-19 patients” by Ricci et al. The authors grouped the patients into two groups according to the vitamin D levels measured at the time of admission into the hospital and reported that lower vitamin D levels are associated with elevated D-dimer and IL-6 levels, low CD4/CD8 ratio and compromised clinical findings with elevated LIPI and SOFA scores. However, review of recent literature shows this association to be debatable. The 25-hydroxyvitamin D levels in the initial phase of critical illness have been reported to drop rapidly and hence consideration of the time of measurement from symptom onset would have enhanced the clinical relevance of these findings. Inferred association between vitamin D levels and disease severity based on SOFA score in COVID-19 patients, needs to be further explored in the light of the recent literature which casts doubt on using SOFA score at admission to predict mortality in COVID-19.

## Letter

We read with great interest the article entitled: “Circulating Vitamin D levels status and clinical prognostic indices in COVID-19 patients” by Ricci et al. [1]. The authors enrolled 52 COVID-19 patients in the study and collected vitamin D levels in the acute phase of the disease and concluded that lower vitamin D levels are associated with elevated D-dimer and Interleukin-6 (IL-6) levels, low CD4/CD8 ratio and compromised clinical findings with elevated Lung Immune Prognosis Index (LIPI) and Sequential Organ Failure Assessment (SOFA) scores.

A recently published metanalysis conducted by Kazemi et al. [2] showed equivocal outcomes of vitamin D deficiency and mortality association and inconsistent results of the association between vitamin D deficiency and pulmonary infiltrates/inflammation in COVID-19. Vitamin D insufficiency is common in hospitalized patients with acute illness especially in critically ill patients [3–5]. Serum 25-hydroxyvitamin D levels measured during the time of acute illness may be an unreliable biomarker of vitamin D status [6]. Thus, the vitamin D levels, that were measured after the onset of acute inflammatory insult in this study by Ricci et al., might reflect the acute phase of the disease rather than true vitamin D levels. Future studies should consider measuring vitamin D levels before the acute insult. If the measurement is done during the acute phase of the illness, consideration should be given to the days from symptom onset at the time of measuring vitamin D levels. Since different patients might present to the hospital at different stages of the acute illness and studies have noted rapid changes in the 25-hydroxyvitamin D levels in the initial phase of critical illness [7].

In addition, a variety of factors might affect vitamin D binding proteins and 25-hydroxyvitamin D levels including race, age, gender, obesity, chronic liver and kidney disease, diabetes and primary hyperparathyroidism [6]. Growing number of observational studies report significant association between preexisting comorbidities and clinical outcomes in COVID-19 patients [8–11]. In the study by Ricci et al., majority of patients were elderly, but consideration to various preexisting comorbidities and heterogeneity of race is missing, which undermines the clinical relevance of the results of this study.

A number of recent studies suggest inflammatory cytokine especially higher IL-6 associated with COVID-19 severity and survival [12–14]. A review of the literature looking at the association between vitamin D deficiency and IL-6 levels offers mixed evidence. Study by Radujkovic et al.[15] noted elevated IL-6 levels among patients with vitamin D deficiency similar to what has been noted by Ricci et al., whereas several other studies report no such association[16–19]. Similar conflicting results have been noted for elevated D-dimer levels, with many studies reporting no statistical significance [16, 19, 20], thereby further research is needed to elucidate this association as noted by the authors. Additionally Ricci et al. noted increased lung lesions in patients with low vitamin D levels similar to the study by Abrishami et al. [21] wherein they noted higher vitamin D levels associated with decreased amount of lung involvement on imaging, whereas Pizzini et al. [20] noted no such association. Ricci et al. also report that the patients with more severe COVID-19 disease had lower Vitamin D plasma levels regardless of age, whereas there is no mention of any subgroup analysis based on age, or the value of interaction between vitamin D levels and age in their statistical model. Hence it might be prudent to exercise caution while interpreting this statement.

We had earlier conducted a study looking at vitamin D levels and clinical outcomes in COVID-19 patients [22], wherein vitamin D levels were classified as ≥ 20 ng/mL (patients with normal vitamin D levels) and < 20 ng/mL (patients with low vitamin D levels). We found no significant association of vitamin D levels with mortality, the need for mechanical ventilation, intensive care unit (ICU) admission, and thromboembolism in COVID-19. Optimal levels of 25-hydroxyvitamin D for bone and general health have not yet been established [23–25] and most experts agree that vitamin D deficiency should be defined as 25-hydroxyvitamin D of < 20 ng/ml [26], it would have been of interest if the authors Ricci et al. had conducted further analysis looking at the results with vitamin D levels < 20 ng/mL and ≥ 20 ng/mL. Our data consisted of COVID-19 positive patients who presented to the hospital and had documented vitamin D, 25-OH level within the past 12 months. In the cohort of our study [22], there were 117 males (43.3%) and 153 females (56.7%). The mean age of patients was 63.81 years (mean ± SD, 14.69). More than half of the patients (n = 139, 51.5%) were in the 65 and older age group, with Blacks being the predominant race (n = 216, 80%). We conducted further exploratory analysis on our data and reclassified vitamin D levels as < 10 ng/mL and ≥ 10 ng/mL (as done by Ricci et al.). Vitamin D level < 10 ng/mL was seen in 39 (14.4%) patients and ≥ 10 ng/mL was seen in 231 (85.6%) patients. After adjusting for age, sex, race, BMI and presence of comorbidities using binary logistic regression, no significant association was seen with mortality (OR = 0.85; 95% CI, 0.33–2.17; p = 0.73), need for ICU admission (OR = 0.95; 95% CI, 0.44–2.06; p = 0.9) or mechanical ventilation (OR = 0.55; 95% CI, 0.20–1.49; p = 0.24). Additionally, no significant difference was seen in laboratory values (within 24 h of admission using Chi square test)- lymphopenia (Absolute lymphocyte counts less than 1000 per microliter) (17 v/s 111 patients, p = 0.57), ferritin > 300 ng/mL (19 v/s 131 patients, p = 0.47) and D-dimer > 2 mg/L (15 v/s 76 patients, p = 0.29), between the two groups (patients with vitamin D levels as < 10 ng/mL, and patients with vitamin D levels ≥ 10 ng/mL). Some of the recent studies note that SOFA score at presentation is less accurate in predicting mortality or the need for mechanical ventilation in COVID-19 patients [27, 28]. In the study by Ricci et al. they note higher SOFA scores in patients with vitamin D levels < 10 ng/mL however no significant association with mortality was noted as acknowledged by the authors, we advise a word of caution since the authors report the association between worse outcomes and low vitamin D levels based on elevated SOFA scores.

Notwithstanding these limitations, Ricci et al. illustrate a relationship between low vitamin D levels and inflammatory markers and radiological findings seen in COVID-19 patients. Consideration to address these limitations in future studies would immensely enhance the clinical relevance of these findings. Ideally, vitamin D levels immediately preceding the acute illness should be used to study the association of vitamin D with disease severity. Large community-based studies and randomized clinical trials of vitamin D supplementation in deficiency patients can unravel the enigma of possible role of vitamin D in the disease progression and severity of symptoms in COVID-19 patients.

## Data Availability

The deidentified data that support the findings of this study can be available from the corresponding author upon reasonable request and appropriate permission from the institutional IRB.

## Abbreviations

IL-6: Interleukin-6
LIPI: Lung Immune Prognosis Index
SOFA: Sequential Organ Failure Assessment
ICU: Intensive care unit
OR: Odds ratio
CI: Confidence interval
SD: Standard deviation
